# Familiarity and task context shape the use of acoustic information in voice identity perception

**DOI:** 10.1016/j.cognition.2021.104780

**Published:** 2021-10

**Authors:** Nadine Lavan, Jens Kreitewolf, Jonas Obleser, Carolyn McGettigan

**Affiliations:** a Department of Biological and Experimental Psychology, School of Biological and Chemical Sciences, Queen Mary University of London, London, United Kingdom; b Department of Speech, Hearing and Phonetic Sciences, University College London, London, United Kingdom; c Department of Psychology, University of Lübeck, Lübeck, Germany; d Department of Psychology, McGill University, Montréal, Canada; e Department of Mathematics and Statistics, McGill University, Montréal, Canada; f Center for Brain, Behaviour, and Metabolism, University of Lübeck, Lübeck, Germany

**Keywords:** Voices, Familiarity, Identity perception, Acoustics

## Abstract

Familiar and unfamiliar voice perception are often understood as being distinct from each other. For identity perception, theoretical work has proposed that listeners use acoustic information in different ways to perceive identity from familiar and unfamiliar voices: Unfamiliar voices are thought to be processed based on close comparisons of acoustic properties, while familiar voices are processed based on diagnostic acoustic features that activate a stored person-specific representation of that voice. To date no empirical study has directly examined whether and how familiar and unfamiliar listeners differ in their use of acoustic information for identity perception. Here, we tested this theoretical claim by linking listeners' judgements in voice identity tasks to complex acoustic representation — spectral similarity of the heard voice recordings. Participants (*N* = 177) who were either familiar or unfamiliar with a set of voices completed an identity discrimination task (Experiment 1) or an identity sorting task (Experiment 2). In both experiments, identity judgements for familiar and unfamiliar voices were guided by spectral similarity: Pairs of recordings with greater acoustic similarity were more likely to be perceived as belonging to the same voice identity. However, while there were no differences in how familiar and unfamiliar listeners used acoustic information for identity discrimination, differences were apparent for identity sorting. Our study therefore challenges proposals that view familiar and unfamiliar voice perception as being at all times distinct. Instead, our data suggest a critical role of the listening situation in which familiar and unfamiliar voices are evaluated, thus characterising voice identity perception as a highly dynamic process in which listeners opportunistically make use of any kind of information they can access.

## 1 Introduction

From merely hearing a voice, listeners can form a wealth of impressions about the person who is speaking: Is the person male or female? Do they sound friendly? Are they familiar? Amongst these impressions, the process of trying to work out exactly whose voice we are hearing — i.e. identity perception — has received substantial attention in the literature (e.g. Kreiman & Sidtis, 2011; Mathias & von Kriegstein, 2014). Within this literature, two broad streams of research have emerged: Studies that consider perception of familiar identities from voices, and studies that examine the perception of unfamiliar voices.

In general, the processes supporting voice identity perception have often been seen as distinct for familiar and unfamiliar individuals (e.g. Stevenage, 2018 for a recent review). This view is based on, and supported by, empirical findings that outline differences between familiar and unfamiliar voice identity perception. Early evidence comes from neuropsychological studies: Here, dissociations between unfamiliar voice discrimination and familiar voice recognition are apparent. For unfamiliar voice discrimination, listeners are presented with a pair of voice recordings and are asked to judge whether the two recordings were produced by the same person or two different people. For voice recognition studies, listeners are presented with a familiar voice and are most frequently asked to identify it as familiar and/or name it. Patients with brain lesions showed selective impairments in either identity discrimination or recognition, leading to the conclusion that the two must be separate abilities (Van Lancker & Kreiman, 1987). Although these results are intriguing, manipulations of familiarity (familiar vs unfamiliar) overlap with changes in the experimental task (recognition vs discrimination) in these studies. This then creates a difficulty in isolating the effect of familiarity.

Recently, familiar and unfamiliar voice perception have been studied and compared within the same experimental task, primarily to investigate the effects of within-person variability on voice identity perception. Within-person variability in voices describes the observation that the sound of a person's voice varies substantially depending on the speaking situation or speaking environment: The voice of the same person will sound very different depending on whether they are having a casual conversation with a friend, shouting to catch someone's attention, or laughing at a joke (Lavan, Burton, Scott, & McGettigan, 2019). Including such within-person variability in experiments of voice perception has been argued to better approximate voice perception outside of the laboratory than using more controlled stimuli (e.g. corpora of read sentences, see also Burton, 2013 for faces).

The experiments that have examined the effects of within-person variability on voice identity perception report striking behavioural differences in how listeners perceive and cope with variability in familiar and unfamiliar voices. In general, within-person variability, introduced through using stimulus sets that include different speaking styles, languages or vocalisation types, poses challenges to voice identity perception (Lavan, Burston, & Garrido, 2019; Lavan, Scott, & McGettigan, 2016; Smith, Baguley, Robson, Dunn, & Stacey, 2019; Stevenage, Symons, Fletcher, & Coen, 2020; Wester, 2012). Familiar listeners, however, have been shown to cope better with such within-person variability than unfamiliar listeners, leading to more accurate identity perception (see Lavan et al., 2016 for a speaker discrimination task).

Further differences in how unfamiliar and unfamiliar listeners perceive voice identity also emerge in voice identity sorting tasks. In these tasks, participants are presented with several variable voice recordings from a small number of identities (typically two) and are asked to sort the voice recordings into perceived identities. Although both listener groups tend to perceive more voices than are actually present, unfamiliar listeners sort voice recordings into many more perceived identities than familiar listeners (Johnson, McGettigan, & Lavan, 2019; Lavan et al., 2019; Lavan, Burston, & Garrido, 2019; Stevenage et al., 2020). Outside of identity perception itself, a body of work furthermore reports enhanced speech comprehension from familiar versus unfamiliar talkers in challenging listening situations (i.e. speech against noise; Kreitewolf, Mathias, & von Kriegstein, 2017; Johnsrude et al., 2013; Nygaard & Pisoni, 1998).

What is it that drives these reported differences in the processing of familiar and unfamiliar voices? Theoretical work has proposed that different mechanisms are at work depending on whether listeners make identity judgements from unfamiliar versus familiar voices (Kreiman & Sidtis, 2011). Listeners who are familiar with a voice are thought to have formed person-specific representations of that voice. Through these representations, listeners can in principle recognise familiar voices as specific individuals (i.e. they can identify the unique owner of that voice). For unfamiliar voices, however, listeners do not have such a person-specific representation. Thus, unfamiliar voices can by definition not be ‘recognised’, although listeners can still discriminate between different unfamiliar identities, as is readily exemplified by voice identity discrimination tasks (e.g. Mühl, Sheil, Jarutytė, & Bestelmeyer, 2018).

This presence or absence of relevant person-specific representations may conceivably result in fundamental differences in how familiar and unfamiliar voices are processed. This then poses the question of how unfamiliar listeners make judgements about identity in the absence of a person-specific representation. In relation to this, empirical and theoretical work has highlighted the role of acoustic properties during identity perception. It is generally accepted that identity perception from familiar and unfamiliar voices alike is linked to the processing of the low-level acoustic properties of the voices: For example, information about the fundamental frequency (F0; perceived as pitch) or the formant frequencies (perceived as changes in voice quality) have been found to be perceptually-salient cues to voice identity for both familiar and unfamiliar voices (Baumann & Belin, 2010; Lavner, Gath, & Rosenhouse, 2000).

While these findings suggest that the kind of acoustic information familiar and unfamiliar listeners use during identity judgements overlaps, theoretical work has proposed that familiar and unfamiliar listeners differ in *how* they use the available acoustic information to make identity judgements. It has been suggested that familiar voices may be processed in a holistic manner, where the processing of certain diagnostic acoustic properties is sufficient to access person-specific representation. Which features are diagnostic for a voice has, however, been noted to be both listener- and voice-dependent (Kreiman & Sidtis, 2011, see also Lavner et al., 2000). Unfamiliar voices, on the other hand, are thought to be processed during identity discrimination via close acoustic comparison of any number of low-level acoustic features (Kreiman & Sidtis, 2011, p 187ff). Similar proposals have been put forward in the face perception literature, where it has been suggested that unfamiliar faces are processed based on comparisons of low-level visual cues, while familiar face processing is thought to be less directly tied to visual image properties (Hancock, Bruce & Burton, 2000).

There is, to date, no comprehensive empirical investigation that directly tests the theoretical proposal that familiar and unfamiliar listeners use acoustic information for identity judgements in a differential manner. In two experiments, we therefore set out to formally examine this research question by directly relating voice identity judgements by both familiar and unfamiliar listeners to the acoustic properties of the perceived voice recordings. We did this by computing spectral representations of the sounds and using these to capture the acoustic similarity between voice recordings. If familiar and unfamiliar listeners use acoustic information differently, the relationship between behavioural voice identity judgements and the acoustic properties of a voice should differ for the two groups of listeners. If listeners use acoustic information in similar ways, we should not observe a difference between listener groups.

In the current study, we used naturally-varying voice stimuli from the TV show *Breaking Bad.* Familiarity with the voices in our study was then manipulated by recruiting participants who had watched the TV show and were thus familiar with the voices, as well as participants who had not watched the show, thus being unfamiliar with the voices. In Experiment 1, groups of such familiar and unfamiliar listeners completed a voice identity discrimination task for two separate stimulus sets. In Experiment 2, we re-analysed data from a previous study using the same two stimulus sets as in Experiment 1, in which a sample of familiar and unfamiliar listeners completed two voice identity sorting tasks (Lavan, Burston, Ladwa, et al., 2019). Discrimination and sorting tasks can be completed by familiar and unfamiliar listeners alike, enabling us to directly compare voice identity judgements made by the two listener groups in the same task. At the same time, these task paradigms differ in how listeners can approach their voice identity judgements. While discrimination tasks rigidly and iteratively present listeners with pairs of voice recordings, leaving relatively little scope in how to engage with the task, sorting tasks do not dictate which recordings are presented and permit listeners to engage with the recordings in a self-directed manner. Critically, however, the inclusion of two different voice identity tasks and two different sets of naturally-varying stimuli in our study design enabled us to explore how any findings may generalize and conceptually replicate across different listening situations and voice recordings.

## 2 Experiment 1: Voice identity discrimination

In our first experiment, we investigated whether the acoustic information of voice recordings informs how listeners perceive identity from naturally-varying stimuli. Specifically, we examined whether familiar and unfamiliar listeners differ in how they use acoustic information to guide their identity discrimination behaviour. For this purpose, listeners were presented with pairs of naturally-varying voice recordings. Participants were then asked to judge whether the pair of voice recordings had been produced by the same person or two different people. To investigate the generalizability of our findings, we ran this study with two separate stimulus sets in two samples of participants.

We then linked listeners' discrimination behaviour to the acoustic properties of the stimuli used. To quantify the acoustic properties of the voice recordings, we computed the spectral energy of the voice stimuli along the frequency range (see Methods for details). Based on these spectral representations of the recordings, we computed the acoustic similarity of all pairs of voice recordings that listeners heard during the speaker discrimination task.

We propose that in the context of naturally-varying voices, using such spectral representations as acoustic measures is preferable to other measures that have frequently been used in voice research, such as the mean F0 and/or measures derived from formants, such as vocal tract length (Baumann & Belin, 2010; Gaudrain, Li, Ban, & Patterson, 2009; Kreitewolf, Mathias, Trapeau, Obleser, & Schönwiesner, 2018; Lavner et al., 2000; see Mathias & von Kriegstein, 2014, for a review). First, the spectral representations we describe are richer signals that include - but are not restricted to - F0 and formant-related acoustic information within the same measure. Second, F0 and formant measures rely on the accurate tracking of these acoustic properties within the voice recordings. However, when using naturally-varying stimuli, voice and recording quality can differ widely, leading to increased measurement error in such tracking procedures. Spectral representations, on the other hand, are based on fast-fourier transforms that take the full acoustic signal into account, thus reducing such measurement error.

We predicted that both listener groups are likely to use acoustic similarity to guide their voice identity judgements, such that acoustically more similar recordings should be more likely to be perceived as coming from the same person. We, however, also predicted that unfamiliar listeners may differ from familiar listeners in how they do this. Since it has been proposed that unfamiliar listeners rely more on close acoustic comparisons during identity perception than familiar listeners (Kreiman & Sidtis, 2011), we specifically predicted that in our experiment unfamiliar listeners should require greater acoustic similarity to perceive different voice recordings as belonging to the same underlying voice identity compared to familiar listeners (independently of the accuracy of the response). That is, if unfamiliar listeners base identity judgements more strongly on comparisons of low-level acoustic properties for naturally-varying voice recordings, they should be more likely to perceive acoustically-dissimilar voice recordings as different identities than familiar listeners. Familiar listeners should be able to better cope with the within-person variability as they are proposed to rely less on low-level acoustic comparison, which may be the result of having access to a mental representation of a familiar voice.

## 3 Methods

### 3.1 Participants

In total, 167 participants were tested for this experiment. All participants were recruited via *Prolific.co*, were aged between 18 and 40 years, had no self-reported hearing difficulties, and had an approval rate on Prolific of over 90%.

We recruited listeners that were selected to be either familiar or unfamiliar with the voices used in the task: Familiarity was established via self-report, such that familiar listeners were defined as those who (i) had watched at least one season of the TV show *Breaking Bad* but (ii) did not remember more than 3 of the specific stimuli used in the experiment*.* Unfamiliar listeners reported to have not seen any episodes of the TV show and did not recognise any of the actors from other shows (e.g. Bryan Cranston as the father from *Malcolm in the Middle*). Listeners were paid at a rate of £7.50 per hour and the study was approved by the local ethics committee.

From this full sample, we excluded 43 participants: 15 unfamiliar listeners recognised one of the voices included in the stimuli; 16 familiar listeners reported to have remembered more than three of the specific voice recordings from watching the TV show; 11 familiar listeners had not watched a full season; and 1 participant failed to pay sufficient attention to the task, indicated by their incorrect responses to more than 20% of the vigilance trials (see Procedure).

This resulted in a final sample of *N* = 121 listeners: 59 familiar listeners, 30 of whom completed the task with voice recordings that were highly expressive (mean age = 27.0 years, SD = 5.6 years, 15 female) and 29 of whom completed the task with low-expressive recordings (mean age = 27.9 years, SD = 5.1 years, 14 female). 62 unfamiliar listeners, 31 of which completed the task with the highly expressive stimuli (mean age = 27.0 years, SD = 6.1 years, 15 female) and 31 of which completed the task with the low-expressive stimuli (mean age = 27.6 years, SD = 6.7 years, 19 female).

### 3.2 Materials

#### 3.2.1 Voice recordings

There were two stimulus sets for this experiment. One stimulus set included “low-expressive” recordings — that is, voice recordings with no pronounced emotional or otherwise expressive content and a largely conversational tone of voice (see Lavan, Burston, Ladwa, et al., 2019). The other stimulus set included voice recordings that were high in vocal expressiveness, such as shouting, growling, and other non-conversational types of speech. The stimulus sets were used opportunistically for this experiment: We were therefore not interested in the effect of expressiveness per se, but included the two stimulus sets to test the generalizability and replicability of our findings across different kinds of naturally-varying voice recordings.

Each stimulus set included 30 short, naturally-varying voice recordings, sampling 15 recordings each from two of the prominent characters of the TV show *Breaking Bad* (Hank Schrader and Walter White). Recordings varied substantially in linguistic content, speaking style, speaking environment, and conversation partners. The recordings ranged between 1.2 and 4 s in duration and contained meaningful utterances (e.g. “You can have any future that you want”, “You're the smartest guy I ever met”), with minimal background noise and no other voices being present in the recording.

The recordings did not include any catchphrases or otherwise diagnostic linguistic information (e.g. referring to a character's name, etc). Stimuli were normalized for peak amplitude (to 0.40 Pa), and low-pass filtered at 10 kHz (using a Hann bandpass filter with upper and lower edges 0 Hz and 10 kHz, smoothing 20 Hz) using Praat (Boersma & Weenink, 2018). Long silences were cut.

### 3.3 Procedure

Familiar and unfamiliar listeners completed a voice identity discrimination task implemented via the Gorilla Experiment Builder (*www.gorilla.sc*; Anwyl-Irvine, Massonnié, Flitton, Kirkham, & Evershed, 2020). After reading the information sheet and giving consent to take part in the study, participants were required to pass a headphone screening to ensure they were able to hear the sounds and that they were using headphones (Woods, Siegel, Traer, & McDermott, 2017). Listeners were then asked to report on whether they had watched the TV show *Breaking Bad*.

After this, listeners were asked to complete a voice identity discrimination task, for which they were randomly assigned to either judge the low-expressive or high-expressive stimuli. A similar number of familiar and unfamiliar listeners was assigned to each stimulus set. After a short practice, each participant completed 145 discrimination trials. From the 30 stimuli, 435 combinations of pairs of stimuli can be created (all possible combinations, where stimulus order does not matter and where pairs that included the same stimulus twice are not included). 435 pairs were, however, deemed to be too many trials for an online experiment. We therefore created 3 sets of 145 stimuli each to form representative subsets of pairs for each of the two stimulus sets (low- and high-expressive recordings). These sets included non-overlapping pairs of sounds. The three subsets were furthermore broadly matched for the number of “same identity” and “different identity” trials per subset (ratios of same to different trials ranged between 1.01 and 1.13 per subset), as well as the number of times a given voice recording was presented within the task. Finally, we ensured that the distribution of acoustic similarities within each subset was comparable to the distribution of the complete set of pairs. The three 3 subsets were counterbalanced across participants within each of the low- and high-expressive stimulus sets respectively. This method of counterbalancing thus ensured that all possible combinations of stimuli were sampled evenly across participants.

For the voice identity discrimination task, listeners were presented with the two voice recordings within a stimulus pair, one after another with an inter-stimulus interval of 500 ms, during which a fixation cross was visible. After the presentation of the second stimulus, participants registered their decision (“same person” or “different people”) via a mouse click. Listeners were not able to revisit trials or replay stimuli and no feedback was given. Twelve catch trials were randomly inserted throughout the task to ensure that listeners were paying attention: For these catch trials, listeners were presented with a single recording of a text-to-speech voice saying either ‘same’ or ‘different’. Listeners were instructed to give the corresponding response for these catch trials.

After completing the voice identity discrimination task, familiar listeners were asked to specify whether they remembered any of the recordings from watching the TV show. Unfamiliar listeners were asked if they recognised any of the voices included in the experiment (see exclusions under Participants).

### 3.4 Data analysis

Data were coded such that pairs of recordings that were perceived to have been produced by the same person were coded as 1 and pairs of recordings that were perceived to belong to different people were coded as 0. We were thus coding raw identity discrimination behaviour – i.e. the probability of labelling a pair as “same person” – as opposed to the accuracy of this identity discrimination.

#### 3.4.1 Calculating acoustic representations of voice recordings

**Fig. 1:**
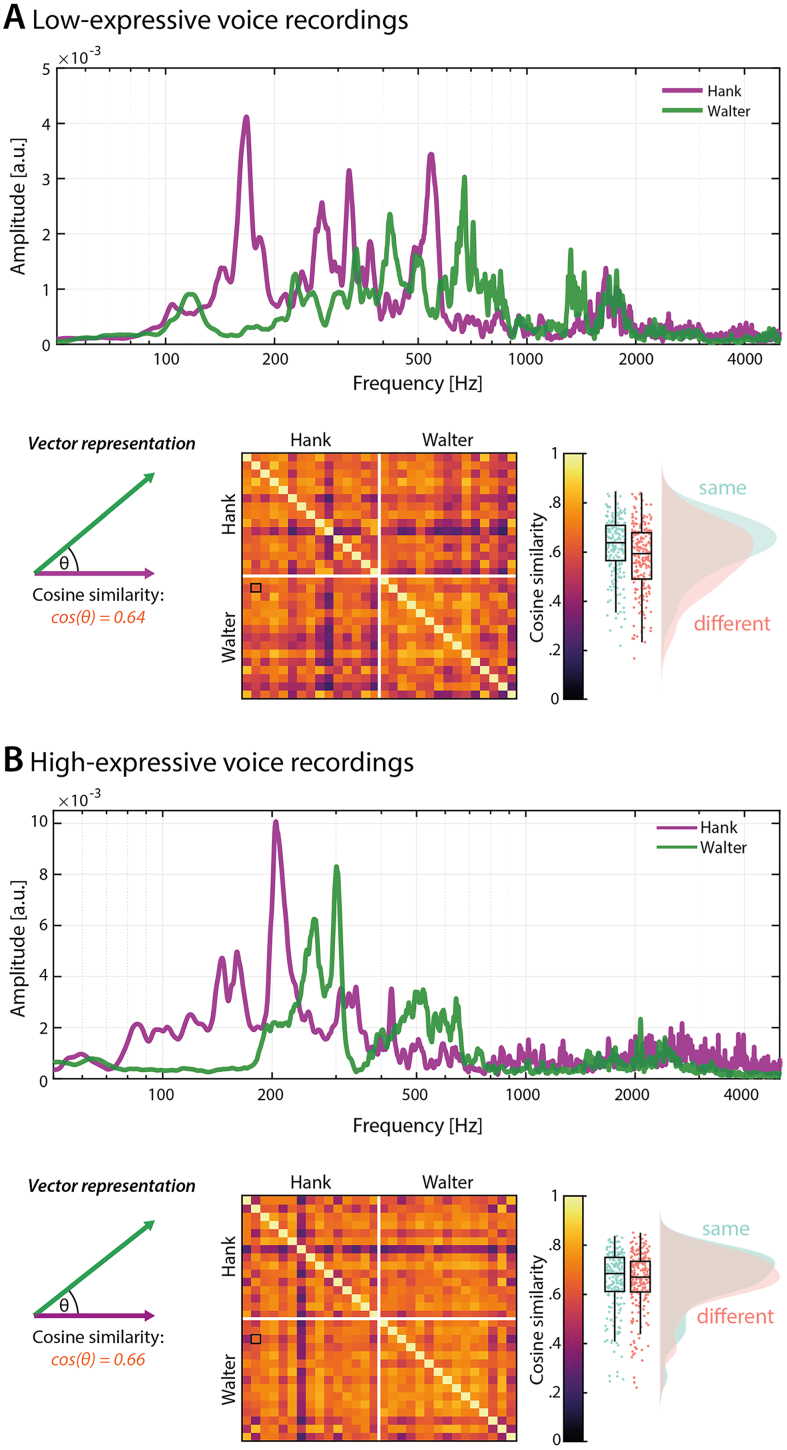
Example spectra and acoustic similarity for (**A**) low- and (**B**) high-expressive voice recordings. *Top*: Spectra of two example sounds spoken by Hank (purple) and Walter (green) shown on a logarithmic frequency axis. *Bottom*: The same spectra shown as normalized vectors. Note that the veridical number of dimensions is defined by the frequency resolution of spectra (see text for details). We calculated cosine similarity for pairs of spectra as a measure of acoustic similarity. Independent of the number of dimensions, cosine similarity is equal to the cosine of the angle between the vectors (θ). The matrix shows cosine similarity for all pairs of spectra, sorted by voice identity. The outlined cell codes the similarity between the two example spectra. Box plots and distributions of cosine similarity are shown separately for pairs of sounds that were produced by the same person (light green) and different people (pink). While the distributions overlap to a great extent, pairs of spectra within the same voice are, on average, more similar than pairs of spectra from different voices. (For interpretation of the references to colour in this figure legend, the reader is referred to the web version of this article.)

To obtain representations of voice recordings, we computed acoustic spectra. These spectra are frequency-resolved representations of spectral energy that capture information about F0, formants, and other peaks of acoustic energy (e.g. harmonics) within a voice recording. Fig. 1 shows examples of such spectra for stimuli used in this study. We reasoned that these acoustic spectra include aspects of the acoustic information that are relevant to identity perception from both familiar and unfamiliar voices (see Baumann & Belin, 2010; Lavner et al., 2000).

To calculate acoustic spectra of individual sounds, we used a fast-fourier transform with a Hann window, where the window length was defined by the length of the sound vector. Since our sounds differed in length, this procedure resulted in spectra with different frequency resolutions. In a following step, we therefore interpolated the resultant frequency vectors to the spectrum of maximal length. We thus obtained the same frequency resolution in all spectra within a given stimulus set (i.e. about 0.38 Hz for all low-expressive and about 0.34 Hz for all high-expressive sounds).

The resultant spectral representations of the voice recordings were then processed further: First, we low-pass filtered spectra using a second-order Butterworth filter with 1 kHz high-frequency cut-off to reduce noise and obtain smoother spectra. Second, we restricted the frequency range to 50–5000 Hz as there was no meaningful voice-related spectral energy below the lower cut-off and only limited voice-related spectral energy above 5000 Hz. This procedure resulted in frequency vectors of length 13,116 for low-expressive and 14,737 for high-expressive sounds.

Using these spectral representations of the voice recordings, we then assessed the acoustic similarity between all pairs of voice recordings presented in this study, using cosine similarity. Acoustic similarity was chosen as a measure to address our research question, because it has been proposed that at least unfamiliar listeners make identity judgements based on comparisons of (low-level or elemental) acoustic features (Kreiman & Sidtis, 2011). We extended this proposal and used acoustic similarity as a way to operationalize these perceptual comparisons described by Kreiman and Sidtis (2011) for the purpose of our study. For the computations of the similarity, we chose cosine similarity over other distance measures (e.g. Euclidean distance) because of the high dimensionality of spectra (see above). Cosine similarity is calculated as the inner product of two normalized frequency vectors. Note that, irrespective of vector length (i.e. number of dimensions), cosine similarity is equal to the cosine of the angle between two vectors. Normalized cosine similarity can range between 0 and 1, with higher values indicating higher similarity (see Fig. 1A and B for cosine similarities across pairs of low- and high-expressive sounds, respectively).

#### 3.4.2 Relating identity discrimination behaviour to acoustic properties

Does acoustic similarity predict individual participants' identity discrimination behaviour (i.e. whether participants perceive two voice recordings to be the same or two different voice identities)? To answer this question, we ran Generalised Linear Mixed Models (GLMM) using the *lme4* package (Bates, Maechler, Bolker, Walker, & Haubo Bojesen Christensen, 2015) in the *R* environment (Rstudio 3.6.1). The binary identity discrimination behaviour per stimulus pair (1 — same identity, 0 — different identities) was entered as the dependent variable into logistic mixed-effects regression models. We ran one model per stimulus set. In these models, familiarity (familiar vs unfamiliar), acoustic similarity, and the interaction of these effects were entered as fixed effects. Acoustic similarity was z-scored to obtain standardized estimates. Familiarity was modelled as a between-subject factor (with unfamiliar as reference level). Participants and the two voice recordings per pair were entered as random intercepts:

$$\text{glmer}(\text{discrimination behaviour}\sim \text{acoustic similarity}*\text{familiarity}+\left(1\;|\;\text{participant}\right)+\left(1\;|\;\text{item1}\right)+\left(1\;|\;\text{item2}\right)$$

Post-hoc tests were conducted using *emtrends* from the *emmeans* package (Lenth, 2017). We applied Tukey's range tests to correct for multiple comparisons where necessary. The significance of fixed effects and interactions were evaluated using *z* tests from within the GLMMs. We compared the results from these *z* tests to significance obtained through likelihood ratio tests (comparing the full model with a reduced model excluding the predictor of interest) and determined that results for both methods were comparable.

We provide odds ratios (ORs) as estimates of effect sizes. An OR of 1 indicates that no effect is present, ORs that deviate from 1 indicate that an effect is present. The larger the deviation, the bigger the effect. For simple effects (e.g. effect of acoustic similarity in a given listener group), we report 95% confidence intervals along ORs and consider a slope to be significant if the confidence interval does not contain 1.

## 4 Results

### 4.1 Task performance

#### 4.1.1 Low-expressive voice recordings

Overall, familiar listeners outperformed unfamiliar listeners (81.2% [SD = 18.0%] vs 68.8% [SD = 19.6%] correct). This replicates familiarity advantages that have been previously reported in voice discrimination tasks (e.g. Lavan et al., 2016). We split up the data set into trials including the same identity and trials including different identities and observed that this familiarity advantage was present in both same (72.5% [SD = 20.4%] vs 57.0% [SD = 19.6%] correct) and different trials (89.9% [SD = 9.5%] vs 80.6% [SD = 10.2%]). All comparisons between familiar and unfamiliar were significant as confirmed by independent-samples Welch's *t*-tests (all *p*s < 0.001).

#### 4.1.2 High-expressive voice recordings

Familiar listeners also outperformed unfamiliar listeners in the stimulus set including highly expressive voice recordings (71.6% [SD = 15.0%] vs 59.6% [SD = 14.2%] correct). As was observed for low-expressive recordings, this familiarity advantage was present in trials including the same identity (69.4% [SD = 13.3%] vs 52.4% [SD = 13.5%]) and trials including different identities (73.6% [SD = 16.5%] vs 66.3% [SD = 11.1%]). All comparisons between familiar and unfamiliar listeners were again significant as confirmed by independent-samples Welch's t-tests (all *p*s < 0.001), with the exception of “different identity” trials (*t*[114.93] = −1.41, *p* = .161).

For both stimulus sets, the low accuracy for unfamiliar listeners on trials including the same identity echoes findings of previous voice identity sorting tasks that have used naturally-varying stimuli (e.g Johnson et al., 2019; Lavan, Burston, & Garrido, 2019; Lavan, Burston, Ladwa, et al., 2019): Unfamiliar listeners show a stronger tendency to perceive within-person variability as different voice identities, thereby systematically failing to label variable recordings as coming from the same person. When perceiving between-person variability in sorting tasks, familiar and unfamiliar listeners behave more similarly – this is also the case for the current discrimination data, where the group difference in accuracy is larger and more consistent for ‘same identity’ trials than for ‘different identity’ trials.

### 4.2 Familiar and unfamiliar listeners use acoustic information in similar ways

#### 4.2.1 Low-expressive voice recordings

**Fig. 2:**
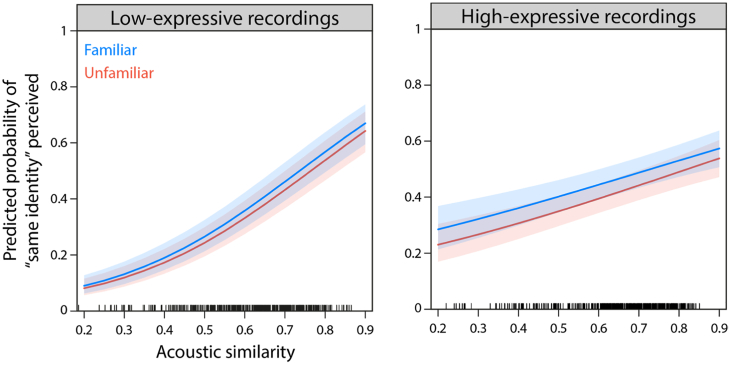
Results of GLMMs for low- (left) and high-expressive voice recordings (right) in the discrimination task. Predicted probabilities of perceiving the same voice identity in pairs of recordings is plotted as a function of acoustic similarity, and separately for familiar (blue) and unfamiliar listeners (orange). These results show that, for both listener groups and stimulus sets, pairs of recordings are more likely to be perceived as the same voice identity if their acoustic properties are more similar. Tick marks above the x-axis show the spread of the cosine similarity values (0 — least similar, 1 — identical) in our data. (For interpretation of the references to colour in this figure legend, the reader is referred to the web version of this article.)

Fig. 2 shows the predicted probabilities of perceiving the same voice identity in pairs of recordings as a function of acoustic similarity.

A logistic mixed-effects regression revealed a significant main effect of acoustic similarity on discrimination behaviour (OR = 1.72, CI[1.58;1.86]; *Z =* 13.14, *p* < .001). The odds ratio indicates that the model predicts an increase in the probability that two voice recordings are perceived as belonging to the same identity by factor 1.72 per unit increase in acoustic similarity. This result therefore demonstrates a strong positive relationship between voice identity perception and acoustic similarity (Fig. 2, left).

The regression analysis did neither reveal a significant main effect of familiarity (OR = 1.13, CI[0.85;1.48]; *Z =* 0.84, *p* = .404) nor a significant interaction between familiarity and acoustic similarity (OR = 1.00, CI[0.91;1.10]; *Z =* 0.041, *p* = .967). That is, the slopes for familiar and unfamiliar listeners did not significantly differ from each other (compare blue vs orange curves in Fig. 2, left). The slopes for both listener groups were significantly positive (Unfamiliar listeners: OR = 1.71, CI[1.58; 1.86]; Familiar listeners: OR = 1.72, CI[1.59; 1.87]), suggesting that both listener groups strongly rely on acoustic similarity for the discrimination of low-expressive voices. Overall, this model accounted for 19.8% of the total variance in the voice discrimination data.

#### 4.2.2 High-expressive voice recordings

A separate logistic mixed-effects regression explored the effects of acoustic similarity and familiarity on the discrimination of high-expressive voice recordings. The results of this analysis conceptually replicate our findings for low-expressive recordings: There was a significant main effect of acoustic similarity on identity discrimination behaviour (OR = 1.28, CI[1.17;1.39]; *Z =* 5.66, *p* < .001), again showing a positive relationship between voice identity perception and acoustic similarity (Fig. 2, right). As for low-expressive stimuli, the slopes for both familiar and unfamiliar listeners were significantly positive (Unfamiliar listeners: OR = 1.28, CI[1.17;1.39]; Familiar listeners: OR = 1.24, CI[1.14;1.35]), suggesting that both listener groups use acoustic similarity for the discrimination of high-expressive recordings.

Neither the main effect of familiarity was si2gnificant (OR = 1.22, CI[0.99;1.50]; *z* = 1.85, *p* = .066) nor was the interaction of familiarity and acoustic similarity (OR = 0.98, CI[0.89;1.07]; *Z =* −0.54, *p* = .591). Overall, this model accounted for 10.0% of the total variance in the discrimination data.

Taken together, the results from Experiment 1 suggest that, in both stimulus sets, familiar and unfamiliar listeners 1) rely on acoustic similarity of voice recordings for identity discrimination and 2) do this in similar ways.

## 5 Discussion

Familiar and unfamiliar listeners completed a voice identity discrimination task on two different stimulus sets that included naturally-varying voice recordings. When making discrimination judgements, as predicted we find that both listener groups make use of the acoustic information as indexed by our measure of acoustic similarity, for both stimulus sets: The more similar the acoustic properties of a pair of voice recordings, the more likely listeners are to judge these two recordings as coming from the same voice identity.

Crucially, however, within both the low- and high-expressive stimulus sets, the response functions were similar for familiar and unfamiliar listeners. This suggests that the acoustic information represented in spectral representations is used to a similar degree by familiar and unfamiliar listeners. Thus, we found no evidence for the prediction that unfamiliar listeners use acoustic information in a different way to familiar listeners.

Despite the similarities in the use of acoustic information for voice identity discrimination between familiar and unfamiliar listeners, we note marked differences in overall task performance between the two listener groups: Familiar listeners were, on average, more accurate in their voice discrimination behaviour than unfamiliar listeners. We therefore argue that this familiarity benefit must be either driven by 1) differences in task strategy or decision making, 2) familiar listeners being able to access other sources of information to aid their identity judgements, such as being able to access a mental representation of the familiar voices or 3) potentially by acoustic (or perceptual) properties of voice recordings that are not captured by the spectral representations we use here (e.g. temporal cues), since the relationship between acoustic similarity and identity judgements was similar for familiar and unfamiliar listeners.

We further propose that the task used here — voice identity discrimination — is a fairly rigid task for familiar and unfamiliar listeners alike: Trials have a fixed structure and responses are dictated by the nature of the task. It is therefore possible that any differences in how familiar and unfamiliar listeners might flexibly use acoustic information for voice identity perception have been obscured by the specific experimental task. In a second experiment, we therefore used another task that measures voice identity perception using relatively fewer constraints — namely, voice identity sorting — to explore whether potential differences between familiar and unfamiliar listeners might be revealed under these altered task conditions.

## 6 Experiment 2: Voice identity sorting

Do familiar and unfamiliar listeners use acoustic information for voice identity judgements in similar ways in a different, less constrained experimental task? To answer this question, we conducted a second experiment in which familiar and unfamiliar listeners completed a voice identity *sorting* task. Listeners can complete these identity sorting tasks in a self-guided manner, freely selecting which voice recordings to play and replay. This enables participants to, for example, listen closely where necessary and to revisit decisions in the process of forming and re-forming clusters. This contrasts with discrimination tasks (see Experiment 1), in which familiar and unfamiliar listeners alike are forced to align with the task instructions, and which might consequently lead them to listen in a particular, and more uniform way. In the second experiment, we used existing perception data from a voice identity sorting paradigm (Lavan, Burston, Ladwa, et al., 2019) and conducted the same analysis as described in Experiment 1 on these data. If the findings from Experiment 1 are generalizable beyond voice identity discrimination, we predicted that familiar and unfamiliar listeners should again use acoustic information in similar ways in the sorting task.

## 7 Methods

The behavioural voice identity sorting data analysed in this experiment were previously published as part of another paper (Lavan, Burston, Ladwa, et al., 2019).

### 7.1 Participants

This experiment includes data from 29 listeners who had watched the TV show *Breaking Bad* (‘familiar listeners’; mean age = 22.52 years, SD = 6.64 years, 21 female) and 27 listeners who had never watched the TV show *Breaking Bad* (‘unfamiliar listeners’; mean age = 20.4 years, SD = 2.26 years, 15 female). Inclusion and exclusion criteria were the same as in Experiment 1. These participants were recruited via social media and the participant pool of the Department of Psychology at Royal Holloway, University of London. Participants were either entered into a prize draw, received course credit or were paid £5 for their participation. The study was approved by the local ethics committee.

### 7.2 Materials

The same voice recordings as in Experiment 1 were used: 30 low-expressive and 30 high-expressive voice recordings from the same two characters of the TV show *Breaking Bad*.

#### 7.2.1 Identity sorting materials

For each stimulus set, the 30 recordings (2 identities [Hank, Walter] x 15 exemplars) were embedded into a separate Microsoft Powerpoint slide. In addition to these recordings, each slide included 2 duplicates of a recording of a synthetic female voice, saying “Hello. My name is Laura”. These recordings were included as attention checks to verify that participants were completing the task correctly (i.e. we checked that they formed a separate identity cluster for the 2 female voice exemplars). Each voice recording was represented by a number on the screen. These numbers were evenly distributed across the slide, with no obvious clusters being apparent at the start of the task (see also e.g. Lavan, Burston, & Garrido, 2019).

### 7.3 Procedure

Participants completed the experiment online via the online testing platform Qualtrics (qualtrics.com). They were asked to complete the two identity sorting tasks, one after the other. The ordering of the tasks (low expressiveness vs high expressiveness) was counterbalanced across participants. Thus, stimulus set was a within-subjects factor in Experiment 2. Crucially, however, no meaningful learning took place across the repeated sorting tasks (see supplementary materials for Lavan, Burston, Ladwa, et al., 2019). Participants thus first downloaded one of the Powerpoint slides and were asked to sort the voice recordings on this slide into clusters, such that each cluster included the recordings produced by a single speaker (i.e. represented a single perceived speaker identity). Clusters were formed by dragging and dropping recordings on the Powerpoint slide. Participants could replay and move the stimuli as many times as they required. After completing the first sorting task, listeners uploaded the sorted Powerpoint slide onto Qualtrics, completed the second identity sorting task in the same manner, and finally completed a number of debrief questions (see Experiment 1).

### 7.4 Data analysis

#### 7.4.1 Processing the identity sorting data

To examine how the different identity clusters were formed and how recordings were sorted together (or apart), we created a list of all possible pairwise combinations of the stimuli included in the sorting task. Through this procedure, we thus aligned the data of the sorting task with the data from the voice identity discrimination task. There were 435 unique pairs of recordings. Based on the sorted Powerpoint slides, these pairs were coded as 1 if the two stimuli were sorted into the same identity cluster and were coded as 0 if they were sorted into different identity clusters. As in Experiment 1, the data therefore describe identity sorting behaviour and do not reflect the accuracy of this sorting behaviour. Statistical models and analyses were identical to the ones used in Experiment 1.

## 8 Results

### 8.1 Task performance

#### 8.1.1 Low-expressive voice recordings

For each participant, we counted how many clusters participants had formed for each of the slides. Here, we include descriptive statistics only, statistical comparisons are reported in Lavan, Burston, Ladwa, et al. (2019). Unfamiliar listeners systematically failed to ‘tell voices together’, such that naturally-varying voice recordings from the same person are perceived as several different people. This leads to unfamiliar listeners perceiving more identities and thus forming many more clusters than the two that are veridically present (Median = 9 identities, range = 4–15). For familiar listeners, this effect is much reduced, although ‘telling together’ errors still frequently occur (Median = 3 identities, range = 2–9). However, familiar and unfamiliar listeners perform with high, and similar, levels of accuracy when dealing with voice recordings from two different people; that is, they are highly accurate at telling different people apart.

#### 8.1.2 High-expressive voice recordings

The same pattern of results was apparent for the high-expressive voice recordings: Unfamiliar listeners formed far more clusters than familiar listeners, reflecting a failure to “tell together” different variable recordings of the same voice (Unfamiliar: Median = 9 identities, range = 3–16; Familiar: Median = 2 identities, range = 2–9). For full results, please see Lavan, Burston, Ladwa, et al. (2019).

### 8.2 Voice familiarity modulates the use of acoustic similarity in identity sorting

#### 8.2.1 Low-expressive voice recordings

**Fig. 3:**
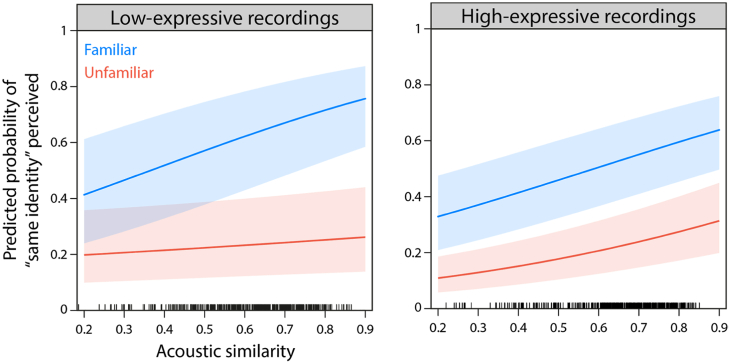
Results of GLMMs for low- (left) and high-expressive voice recordings (right) in the voice sorting task. Predicted probabilities of perceiving the same voice identity from pairs of recordings plotted as a function of acoustic similarity, and separately for familiar (blue) and unfamiliar listeners (orange). While the overall results suggest a relationship between acoustic similarity and voice sorting, this relationship depends on voice familiarity: Familiar listeners seem to rely more on acoustic similarity for voice sorting than unfamiliar listeners (see text for details). Tick marks above the x-axis show the spread of the cosine similarity in our data. (For interpretation of the references to colour in this figure legend, the reader is referred to the web version of this article.)

Fig. 3 shows the predicted probabilities of perceiving the same voice identity in pairs of recordings as a function of acoustic similarity.

A logistic mixed-effects regression revealed a significant interaction of familiarity and acoustic similarity (OR = 1.22, CI[1.14;1.32]; *Z =* 5.36, *p* < .001), suggesting that familiar and unfamiliar listeners differ in their use of acoustic similarity in voice identity sorting. This difference is apparent in the predicted probabilities of perceiving the same voice identity in pairs of recordings (compare blue and orange curves in Fig. 3, left) with a significantly positive slope for familiar listeners (OR = 1.31; CI[1.23;1.38]) and a shallower, non-significant slope for unfamiliar listeners (OR = 1.06; CI[0.99;1.14]).

In addition to the significant interaction, we also found a significant main effect of familiarity in this analysis (OR = 5.79, CI[4.00;8.40]; *Z =* 9.28, *p* < .001), suggesting that familiar listeners were overall more likely to sort pairs of voice recordings into the same identity cluster. The main effect of acoustic similarity remained at trend level (OR = 1.07, CI[1.00;1.14]; *Z =* 1.92, *p* = .055). Overall, this model accounted for 62.1% of the total variance in the sorting data.

#### 8.2.2 High-expressive voice recordings

We again computed a separate logistic mixed-effects regression to explore the effects of familiarity and acoustic similarity on voice identity sorting using high-expressive voice recordings. The results of this analysis also showed marked differences in voice identity sorting between familiar and unfamiliar listeners: There was a significant main effect of familiarity (OR = 3.89, CI[2.66;5.70]; *Z =* 6.98, *p* < .001), again showing that familiar listeners were overall more likely to sort two voice recordings into the same identity cluster. For high-expressive recordings, this effect was however largely independent from acoustic similarity as we found no evidence for an interaction of familiarity and acoustic similarity (OR = 1.00, CI[0.93;1.07]; *Z =* −0.12, *p* = .904).

There was also a significant main effect of acoustic similarity (OR = 1.26, CI[1.17;1.36]; *Z =* 6.19, *p* < .001). The odds ratio of 1.26 indicates a positive relationship between acoustic similarity and voice identity sorting (Fig. 3, right) with significantly positive slopes in both familiar (OR = 1.26, CI[1.18;1.34]) and unfamiliar listeners (OR = 1.26, CI[1.17;1.36]). Overall, this model accounted for 46.8% of the total variance in the sorting data.

In sum, the results for voice identity sorting reveal differences between familiar and unfamiliar listeners. For the sorting of low-expressive voice recordings, we found that the use of acoustic similarity is modulated by voice familiarity: Familiar listeners seem to rely more on acoustic similarity to sort two voice recordings into the same identity cluster. For the sorting of high-expressive recordings, familiar listeners were more likely to sort two voice recordings into the same identity cluster, independent of acoustic similarity. Across all listener groups, tasks, and stimulus sets, our results show a strong positive relationship between voice identity judgements and acoustic similarity in pairs of recordings (except for identity sorting of low-expressive voice recordings in unfamiliar listeners; see orange curve in Fig. 3, left). Critically, however, across the two tasks (voice discrimination and voice sorting), we found marked differences in the use of acoustic similarity by familiar and unfamiliar listeners.

### 8.3 Task effects on the use of acoustic similarity in familiar and unfamiliar listeners

To formally assess whether the task that listeners performed in Experiment 1 (voice discrimination) and Experiment 2 (voice sorting) had a significant effect on the use of acoustic similarity for identity judgements by familiar and unfamiliar listeners, we combined the data from these two experiments and ran two GLMMs, one for each of the two stimulus sets: These GLMMs had the same random structure as the models reported above, with experiment (discrimination vs sorting) added as a fixed factor in addition to the existing fixed effects familiarity and acoustic similarity.

$$\text{glmer}(\text{discrimination behaviour}\sim \text{acoustic similarity}*\text{familiarity}*\text{experiment}+\left(1|\text{participant}\right)+\left(1|\text{item1}\right)+\left(1|\text{item2}\right)$$

For low-expressive voice recordings, the model output for all fixed-effects terms is shown in Table 1. Overall, this model accounted for 36.8% of the total variance in the data. The model included a significant interaction between acoustic similarity, familiarity, and experiment (OR = 0.85, CI[0.75;0.96], *Z* = −2.69, *p* = .007). This three-way interaction statistically confirms our observation that the tasks used in the two experiments had a significant effect on how familiar and unfamiliar listeners use acoustic similarity for voice identity judgements: For voice identity discrimination (Experiments 1), post-hoc tests revealed no significant difference in the use of acoustic similarity between the two listener groups (OR = 0.99, CI[0.90;1.09], *Z* = −0.12, *p* = .99), with significantly positive relationships between acoustic similarity and (predicted) voice identity perception in both familiar (OR = 1.83, CI[1.70;1.97]) and unfamiliar listeners (OR = 1.84, CI[1.71;1.98]; compare Fig. 2, left). For voice identity sorting (Experiment 2), however, post-hoc tests revealed a significant difference in the use of acoustic similarity between listener groups (OR = 1.17, CI[1.09;1.25], *Z* = 4.53, *p* < .001) with a significantly stronger positive relationship between acoustic similarity and (predicted) voice identity perception in familiar (OR = 1.55, CI[1.48;1.63]) compared to unfamiliar listeners (OR = 1.32, CI[1.25;1.41]).

**Table 1:** Model outputs for the GLMM assessing task effects between Experiments 1 and 2 for low-expressive voices. For factors, the reference level is shown in brackets. Please refer to the text for further details.

	Odds ratio	95% CI	*z*	*p*
(Intercept)	0.21	0.14–0.31	−7.75	**<0.001**
Acoustic similarity	1.33	1.25–1.41	9.45	**<0.001**
Familiarity [unfamiliar]	3.86	3.30–4.51	16.98	**<0.001**
Experiment [discrimination]	2.15	1.84–2.52	8.49	**<0.001**
Acoustic similarity * Familiarity	1.17	1.09–1.25	4.53	**<0.001**
Acoustic similarity * Experiment	1.38	1.27–1.51	7.19	**<0.001**
Familiarity * Experiment	0.23	0.19–0.29	−12.99	**<0.001**
Acoustic similarity * Familiarity * Experiment	0.85	0.75–0.96	−2.69	**0.007**

For high-expressive voice recordings, the model output for all fixed-effects terms is reproduced in Table 2. Overall, this model accounted for 28.5% of the total variance in the data. Here, we found no evidence for a task effect (comparison across the two experiments) on the use of acoustic similarity by familiar and unfamiliar listeners: The three-way interaction between acoustic similarity, familiarity, and experiment remained non-significant (OR = 0.98, CI[0.87;1.10], *Z* = −0.38, *p* = .703). The model, however, revealed a significant two-way interaction of familiarity and experiment (OR = 0.28, CI[0.21;0.37], *Z* = −8.89, *p* < .001). Post-hoc tests showed no significant effect of voice familiarity on identity perception in the discrimination task (Experiment 1) (OR = 1.12, CI[0.91;1.39], *Z* = 1.11, *p* = .685). Specifically, the model predicts that both familiar (OR = 0.70, CI[0.51;0.97]) and unfamiliar listeners (OR = 0.62, CI[0.46;0.86]) are overall more likely to perceive different voice identities in pairs of recordings. However, there was a significant effect of voice familiarity on identity perception in the sorting task (Experiment 2; OR = 4.07, CI[3.30;5.02], *Z* = 13.18, *p* < .001): The model predicts that familiar listeners are more likely to perceive the same voice identity in pairs of recordings (OR = 0.82, CI[0.60;1.12]) than unfamiliar listeners (OR = 0.20, CI[0.15;0.28]). In sum, this model therefore formally confirms that there is a significant main effect of voice familiarity for identity sorting but not for discrimination.

**Table 2:** Model outputs for the GLMM assessing task effects between Experiments 1 and 2 for high-expressive voice recordings. For factors, the reference level is shown in brackets. Please refer to the text for further details.

	Odds ratio	95% CI	*z*	*p*
(Intercept)	0.20	0.14–0.27	−9.92	**<0.001**
Acoustic similarity	1.30	1.21–1.38	7.77	**<0.001**
Familiarity [unfamiliar]	4.08	3.31–5.03	13.18	**<0.001**
Experiment [discrimination]	3.11	2.59–3.74	12.17	**<0.001**
Acoustic similarity * Familiarity	0.99	0.92–1.06	−0.37	0.71
Acoustic similarity * Experiment	0.94	0.86–1.02	−1.46	0.145
Familiarity * Experiment	0.28	0.21–0.37	−8.89	**<0.001**
Acoustic similarity * Familiarity * Experiment	0.98	0.87–1.10	−0.38	0.703

Both models therefore indicate that the task used in the different experiments had significant effects on how familiar and unfamiliar listeners perceive voice identities. For low-expressive voice recordings, we found a significant task difference in how familiar versus unfamiliar listeners use acoustic similarity for voice identity judgements. For high-expressive recordings, the effect of voice familiarity on identity perception was modulated by the experimental task independently of acoustic similarity.

## 9 Discussion

As in Experiment 1, our results broadly indicate that acoustic similarity guides listeners' identity judgements: The more similar two voice recordings are in their acoustic properties, the more likely listeners are to sort these two recordings into the same cluster, i.e. perceive them as coming from the same voice identity. In contrast to our findings from Experiment 1, however, we now find significant differences in how familiar and unfamiliar listeners use acoustic information to make identity judgements in the voice sorting task, an effect that manifests in different ways across the two stimulus sets.

For low-expressive voice recordings, the slope describing the relationship between acoustic similarity and sorting behaviour is shallower for unfamiliar listeners compared to familiar listeners (Fig. 3, left). Unfamiliar listeners thus appear to need more acoustic similarity to perceive two voice recordings as the same voice identity. In fact, even for the recordings that are most similar to one another in terms of their acoustic properties in this stimulus set, unfamiliar listeners are, on average, predicted to only perceive these pairs of recordings as the same voice identity some of the time (see orange curve in Fig. 3, left). Unfamiliar listeners thus appear to be biased to perceive any degree of acoustic dissimilarity as evidence of different voice identities being present for this particular stimulus set (see also Lavan, Burston, & Garrido, 2019; Lavan, Burston, Ladwa, et al., 2019). Finding no significant relationship between acoustic similarity and voice identity judgements for the low-expressive stimuli may thus suggest that unfamiliar listeners' sorting behaviour may in this context not be primarily guided by the acoustic properties captured in the acoustic spectra. Alternatively, listeners may be using other properties to guide their sorting behaviour for this particular combination of stimuli, listeners, and experimental task. This lack of a relationship between acoustic similarity and identity judgements stands out within our study. In the context of our data, as well as previous theoretical and empirical work on unfamiliar voice identity perception, this is therefore a surprising finding. Since most previous work on voice identity perception has used low-expressive (but also carefully normed) voice recordings, we would have assumed to replicate findings showing that unfamiliar listeners heavily rely on the comparison of acoustic features, such as F0 and formant-related information, that is encoded in our measure of acoustic similarity. We speculate that higher-order decision making processes may unexpectedly have attenuated or overridden the influence of low-level acoustic properties captured in the acoustic similarity measure. Alternatively or additionally, sorting behaviour may have been more strongly guided by other acoustic properties that are not captured in the spectral representations used here (e.g. temporal features). How this finding has arisen, and why it is only present in this particular set of circumstances can, however, not readily be determined from our existing data.

For high-expressive recordings, we also found differences for familiar and unfamiliar listeners. Here, however, the steepness of the slopes was comparable for listener groups across the range of acoustic similarities. As such, the differences between familiar and unfamiliar listeners were characterized by an overall displacement of the slopes (Fig. 3, right), suggesting that unfamiliar listeners require overall more acoustic similarity to start judging two voice recordings as the same person.

Overall, however, the results of our experiments indicate that under some circumstances familiar and unfamiliar listeners do indeed make differential use of acoustic properties when making identity judgements. We show that the choice of the experimental task had a significant effect on how familiar and unfamiliar listeners use acoustic information to inform voice identity judgements. We propose that the use of a less constrained voice identity task paradigm in Experiment 2 (as compared to Experiment 1) may have created a listening situation in which familiar and unfamiliar consequently use acoustic information in voice identity perception in different ways. As such, sorting tasks may enable familiar listeners to fully capitalize upon their ability to recognize the voices, which is not possible for unfamiliar listeners.

## 10 General discussion

In two experiments, we set out to test whether familiar and unfamiliar listeners differ in the way they use acoustic information in voices when making identity judgements. For this purpose, we introduced spectral representations of stimuli as a novel way to assess acoustic similarity of voices during identity perception. Across two tasks (voice identity discrimination and voice identity sorting) and across two stimulus sets (low-expressive and high-expressive voice recordings), we found evidence linking both familiar and unfamiliar listeners' voice identity judgements to the acoustic information in the stimuli. Acoustic similarity significantly predicted identity perception behaviour, such that more similar voice recordings were overall more likely to be perceived as the same voice identity. This was true in all but one data set (unfamiliar listeners for low-expressive stimuli in Experiment 2). Finding that acoustic information informs voice identity judgements echoes a wealth of previous research linking acoustic information, such as F0 and measures derived from formants, to voice identity perception (Baumann & Belin, 2010, Lavner et al., 2000, see Kreiman & Sidtis, 2011 for a review).

Additionally, we found that how familiar and unfamiliar listeners use acoustic information to make identity judgements depends on the context in which such judgements are required, i.e. in our study, the specific experimental task. For voice identity sorting, the use of acoustic information for voice identity sorting differed depending on the listeners' familiarity with these voices, such that unfamiliar listeners overall required more acoustic similarity to perceive two voices as one identity compared to familiar listeners. However, the use of acoustic information was similar across familiar and unfamiliar listeners for voice identity discrimination.

We speculate that this task dependence is related to the perceptual strategies listeners were able to use to complete the two tasks, such that the available (acoustic) information may have been weighted and used in different manners. For voice identity sorting, listeners can freely choose both a listening strategy and more generally a strategy to complete the task: Listeners can select which voice recordings to listen to (be that through iterative pairwise comparisons or less structured approaches) and how often, and can additionally freely revise perceptual decisions. For voice identity discrimination, however, choices in strategies are limited by the rigid manner of stimulus presentation in the task: Stimulus order is not chosen by the participant; stimuli cannot be repeated; decisions cannot be revised; and, crucially, pairwise presentations and judgements are dictated by the task. This increased flexibility in how a participant can approach voice identity sorting tasks may leave more scope for familiar and unfamiliar listeners to use different strategies from one another in the sorting task. Similarly, for the speaker discrimination task, specific instructions were given to listeners, asking them to determine whether two voice recordings included the same identity or two different identities, while for the sorting task listeners were simply asked to “sort voice recordings by identity”. These differences in the nature of the task and the specificity of instructions could then lead to different listening strategies for the two experiments (for familiar and unfamiliar listeners alike). Existing evidence for such differences in listening strategies can be found in research that shows there is no clear relationship between unfamiliar listeners' task performance for discrimination and sorting tasks (Johnson et al., 2019).

Although to our knowledge no such previous evidence for differences exists for familiar listeners, such differences in listening strategies may in principle also be apparent for this group of listeners. While it is unclear from the literature and our data how the listening strategies for the listener groups specifically differ between sorting and discrimination tasks, we can speculate about the potential nature of strategies: For example for sorting tasks, unfamiliar listeners may iteratively compare a number of individual recordings to each other to establish whether they think these recordings were produced by the same or different identities. Familiar listeners on the other hand may simply listen to individual voice recordings and complete the sorting task primarily via recognizing the specific identity of the voice in the recording. Through the often successful recognition of the identity, familiar listeners can then simply sort the recognized recording into a relevant identity cluster (either “Hank” or “Walt” in our study) alongside (single-item) clusters for any un-recognized recordings. In the sorting task, the proposed strategies for unfamiliar and familiar listeners thus differ substantially from one another, tying in with the differences in the use of acoustic properties that we found for this task. For discrimination tasks, we propose that a different profile of listening strategies may have occurred, in which unfamiliar and familiar listeners behave more similarly to each other. As in the sorting task, unfamiliar listeners might still engage in a process of comparing pairs of recordings to one another. However, unfamiliar listeners are now forced into a strict pairwise discrimination process as opposed to potentially *n*-wise discrimination for the sorting task. Familiar listeners may also be less focussed on recognition of the identities as they are now forced to perceive two (and only two) recordings in relation to each other per trial. While recognition can still play a role, the forced pairwise judgements may be underpinned by listening strategies that may be more similar to the low-level acoustic comparisons that unfamiliar listeners might use during both tasks, and during voice identity perception in general. This relatively greater similarity in perceptual strategies for familiar and unfamiliar listeners may therefore result in less obvious differences in how acoustic similarity is used in our voice discrimination experiment.

Based on our findings, we therefore argue that our study puts into context theoretical claims that view familiar and unfamiliar voice perception as distinct processes (e.g. Kreiman & Sidtis, 2011; Stevenage, 2018): When considering the use of acoustic information during identity perception, as we have done in the current study, no absolute statements about similarities and differences of behaviours can be made for familiar and unfamiliar listeners. Instead, our study suggests that how similarly or dissimilarly familiar and unfamiliar listeners behave varies depending on the stimuli being heard and the overall listening context. Our findings thus show that voice identity processing for familiar and unfamiliar listeners alike may be a process in which the information available to listeners is used in a situation-dependent manner. This then characterizes voice identity perception as a fluid and highly dynamic process, where in addition to some core mechanisms (e.g. low-level comparison of acoustic properties for unfamiliar listeners, identity recognition for familiar listeners) any number of strategies are employed to achieve a perceptual goal by both familiar and unfamiliar listeners (see also Kreiman & Sidtis, 2011). These strategies may at times overlap for the two listeners groups (here: voice discrimination), and may at other times be fairly distinct (here: voice sorting).

In contrast to previous studies (Baumann & Belin, 2010; Lavner et al., 2000) that have related single acoustic features (e.g., F0 and vocal tract length) to voice identity perception, we used more complex acoustic representations of the voice recordings here. We were thus able to capture similarities in rich acoustic information within a single similarity measure. While this approach allowed us to reveal differences between familiar and unfamiliar listeners in their use of acoustic information for voice identity sorting, it remains unclear which acoustic information listeners specifically use in voice perception: Even if familiar and unfamiliar listeners use the rich acoustic information in voices to a similar degree overall — as our study indicates for voice discrimination (Experiment 1) — might there be systematic differences in *how* acoustic features are weighted and processed further? The differences in the overall task performance for familiar and unfamiliar listeners in both experiments suggest that this might indeed be the case. At the same time, it is also worth considering that our findings may change when using different acoustic measures: Although the spectral representations used here are information-rich and capture key acoustic information such as the fundamental frequency and formant characteristics, they do not capture all information that may be relevant to listeners during identity perception (e.g. temporal cues like speech rate). In an alternative analysis approach that would aim at explaining a maximal amount of variance in the data by adding a large number of acoustic predictors to the statistical models. In such a model, we may find that some acoustic predictors are used differentially by familiar and unfamiliar listeners while there is no difference for other acoustic predictors and that these relationships may change depending on the stimuli and/or the experimental task used. Although this approach may in principle shed some light on which acoustic properties may be differentially important for identity perception for the different listener groups, the findings would overall lead to the same overall conclusions that we draw from our findings: Voice identity perception by familiar and unfamiliar listeners is underpinned by a variable set of listening strategies that make differential use of the low-level acoustic properties of the voices heard.

Finally, our findings are likely to be relevant beyond voice identity perception: For example, a large body of literature suggests that voice identity perception and speech comprehension are fundamentally linked by showing enhanced intelligibility of speech produced by a familiar versus unfamiliar voice (e.g., Johnsrude et al., 2013; Kreitewolf et al., 2017; Newman & Evers, 2007; Nygaard & Pisoni, 1998). It has been recently argued that such effects of voice familiarity on speech comprehension are due to improved acoustic representations of familiar voices (Kreitewolf, Wöstmann, Tune, Plöchl, & Obleser, 2019). The current findings may be compatible with this view by suggesting that familiarity indeed affects how listeners interact with the acoustic properties of voices. However, further research is needed to fully explore whether and how such potential differences in familiarity and voice identity representations can be linked to effects of voice familiarity beyond identity perception.

## Appendix A Supplementary data

Supplementary material 1 Key for variable names for the supplementary data. Supplementary material 1
